# RETRACTION: AK4 Promotes the Progression of HER2-Positive Breast Cancer by Facilitating Cell Proliferation and Invasion

**DOI:** 10.1155/dim/9862076

**Published:** 2025-05-07

**Authors:** Disease Markers

RETRACTION: J. Zhang, Y.-T. Yin, C.-H. Wu, R.-L. Qiu, W.-J. Jiang, X.-G. Deng, and Z.-X. Li. “AK4 Promotes the Progression of HER2-Positive Breast Cancer by Facilitating Cell Proliferation and Invasion,” *Disease Markers*, no. 2019 (2019): 1–9. https://doi.org/10.1155/2019/8186091.

The above article, published online on 20 November 2019 in Wiley Online Library (wileyonlinelibrary.com), has been retracted by John Wiley & Sons Ltd.

The retraction has been agreed following an investigation of the concerns raised by *Hoya camphorifolia* and *Phyllosticta caprifolii* on PubPeer [1], which identified multiple instances of inappropriately overlapping figures.

Specifically:

  -Figure 2b: The Immunoblot assay bands corresponding to AK4 and *β*-actin expression in the MCF7 cells have been duplicated in Figure 3b (right) of [2], and Figure 3d (left) of [3], while labelled as other proteins identified in other cell types.
  -Figure 3a: The image of the cell cultures is identical to the culture plates shown in Figure 4a in [4], where the colonies are labelled as different cell types.
  -Figure 3c: Multiple duplications of the 3 wound closure assay images (except the image on the top right) across several publications. The top left panel can be seen in Figure 3c (bottom left) of [5], while the bottom panels are duplicated in Figure 3c (bottom) of [6].
  -Figure 3d: The bottom right section of the MCF7 shRNA image is identical to the bottom left section of the MDA-MB-231 shRNA image.
  -Figure 4a: The tumors are present in Figure 4a of [7] as the 8 tumors on the right side of the figure. Some of these tumors can also be seen in Figure 5 of [4, 8] and [9] in a different arrangement.
  -Figure 4b: The images of the 2 tumors have been duplicated in Figure 4b of [5].
  -Figure 4c: A duplicate of the immunohistochemistry image on the right has been found in Figure 4c (right) of [10] and Figure 5b (left) of [11]. The panel on the left has also been duplicated in [10] as Figure 4d (left). All the figures above attribute the immunohistochemistry images to different tissue types.

As a result of the investigation, the data and conclusions of this article are considered unreliable.

The authors were informed of the decision to retract the paper but did not provide a response.

## References

[1] Hoya camphorifolia and Phyllosticta caprifolii AK4 Promotes the Progression of HER2-Positive Breast Cancer by Facilitating Cell Proliferation and Invasion. *PubPeer*.

[2] Wang L., Zhang X., Liu J., Liu Q. (2022). RETRACTED ARTICLE: Kinesin Family Member 15 Can Promote the Proliferation of Glioblastoma. *Mathematical Biosciences and Engineering: MBE*.

[3] Sun Y.-F., Wu H.-L., Shi R.-F., Chen L., Meng C. (2020). KIF15 Promotes Proliferation and Growth of Hepatocellular Carcinoma. *Analytical Cellular Pathology*.

[4] Gao Z.-A., Yu F., Jia H.-X., Ye Z., Yao S.-J. (2020). ASPM Predicts Poor Prognosis and Regulates Cell Proliferation in Bladder Cancer. *The Kaohsiung Journal of Medical Sciences*.

[5] Cui L., Zhang J.-Y., Ren Z.-P., Zhao H.-J., Li G.-S. (2019). APLNR Promotes the Progression of Osteosarcoma by Stimulating Cell Proliferation and Invasion. *Anti-Cancer Drugs*.

[6] Wang Z.-X., Ren S.-C., Chang Z.-S., Ren J. (2020). Identification of Kinesin Family Member 2A (KIF2A) as a Promising Therapeutic Target for Osteosarcoma. *BioMed Research International*.

[7] Tian D.-W., Wu Z.-L., Jiang L.-M., Gao J., Wu C.-L., Hu H.-L. (2019). KIF5A Promotes Bladder Cancer Proliferation In Vitro and In Vivo. *Disease Markers*.

[8] Li G., Xie Z.-K., Zhu D.-S., Guo T., Cai Q.-L., Wang Y. (2019). KIF20B Promotes the Progression of Clear Cell Renal Cell Carcinoma by Stimulating Cell Proliferation. *Journal of Cellular Physiology*.

[9] Gao C.-T., Ren J., Yu J., Li S.-N., Guo X.-F., Zhou Y.-Z. (2020). KIF23 Enhances Cell Proliferation in Pancreatic Ductal Adenocarcinoma and Is a Potent Therapeutic Target. *Annals of Translational Medicine*.

[10] Qi Z.-Y., Wang F., Yue Y.-Y. (2019). RETRACTED ARTICLE: CYPA Promotes the Progression and Metastasis of Serous Ovarian Cancer (SOC) in Vitro and in Vivo. *Journal of Ovarian Research*.

[11] Sun J.-J., Li H.-L., Ma H., Shi Y., Yin L.-R., Guo S.-J. (2019). SMYD2 Promotes Cervical Cancer Growth by Stimulating Cell Proliferation. *Cell & Bioscience*.

